# Long-Term Exposure to Primary Traffic Pollutants and Lung Function in Children: Cross-Sectional Study and Meta-Analysis

**DOI:** 10.1371/journal.pone.0142565

**Published:** 2015-11-30

**Authors:** Francesco Barone-Adesi, Jennifer E. Dent, David Dajnak, Sean Beevers, H Ross Anderson, Frank J. Kelly, Derek G. Cook, Peter H. Whincup

**Affiliations:** 1 MRC-PHE Centre for Environment and Health, Population Health Research Institute, St George’s, University of London, London, United Kingdom; 2 MRC-PHE Centre for Environment and Health, King’s College London, London, United Kingdom; University of Tennessee Health Science Center, UNITED STATES

## Abstract

**Background:**

There is widespread concern about the possible health effects of traffic-related air pollution. Nitrogen dioxide (NO_2_) is a convenient marker of primary pollution. We investigated the associations between lung function and current residential exposure to a range of air pollutants (particularly NO_2_, NO, NOx and particulate matter) in London children. Moreover, we placed the results for NO_2_ in context with a meta-analysis of published estimates of the association.

**Methods and Findings:**

Associations between primary traffic pollutants and lung function were investigated in 4884 children aged 9–10 years who participated in the Child Heart and Health Study in England (CHASE). A systematic literature search identified 13 studies eligible for inclusion in a meta-analysis. We combined results from the meta-analysis with the distribution of the values of FEV_1_ in CHASE to estimate the prevalence of children with abnormal lung function (FEV_1_<80% of predicted value) expected under different scenarios of NO_2_ exposure. In CHASE, there were non-significant inverse associations between all pollutants except ozone and both FEV1 and FVC. In the meta-analysis, a 10 μg/m^3^ increase in NO_2_ was associated with an 8 ml lower FEV_1_ (95% CI: -14 to -1 ml; p: 0.016). The observed effect was not modified by a reported asthma diagnosis. On the basis of these results, a 10 μg/m^3^ increase in NO_2_ level would translate into a 7% (95% CI: 4% to 12%) increase of the prevalence of children with abnormal lung function.

**Conclusions:**

Exposure to traffic pollution may cause a small overall reduction in lung function and increase the prevalence of children with clinically relevant declines in lung function.

## Introduction

Ambient concentrations of urban air pollution comprise a variety of particulate and gaseous pollutants emanating from different sources. Of particular concern in modern cities is pollution from traffic sources, and its possible effects on respiratory health in children [1–3]. Reduced lung function is a recognized risk factor for long-term cardiovascular and respiratory mortality [4]. However, the association between exposure to air pollution and lung function in children is still uncertain. A review by Gotschi and colleagues suggested small adverse effects of outdoor air pollution on lung function in children [5], but did not provide any quantitative estimate for the effects of individual pollutants. More recently a pooled analysis of five studies, part of the ESCAPE project, found that air pollution was associated with small decreases in lung function [6]. However, other authors reported inconclusive results [7–9].

Arising from a common source, primary traffic pollutants tend to be highly correlated which makes it difficult to separate the effects of individual pollutants. Until recently, the pollutant of most concern has been particulate matter, itself comprising a complex physicochemical mixture but there is now concern that nitrogen dioxide (NO_2_), previously considered to be merely an indicator of more toxic correlated pollutants, may also have a direct toxic effect on lung tissue [3,10]. Regardless of its possible causal role, NO_2_ is one of the most commonly reported markers of traffic-related air pollution in epidemiological studies [11]. For this reason, NO_2_ can be useful to evaluate the health effects of traffic pollution and possible abatement strategies [11].

The Traffic Pollution and Health in London (TRAFFIC) project comprises a collaborative investigation into the effects of air pollution and noise on health outcomes ranging across the whole life-span in the London population. Exposure was estimated using fine scale (20x20m) spatial dispersion models of annual average concentrations of both primary traffic pollutants (pollutants which are directly emitted by traffic) and regional/urban background pollutants (pollutants which are created by both dispersion of the traffic pollutants emitted in the city, other sources in the city and by regional concentrations). Here we report on an investigation linking air pollution exposures with lung function in a large cross-sectional study of lung function in 9–10 year-old children living in London, The Child Heart and Health Study in England (CHASE). The primary hypothesis concerns the effects of near-traffic primary pollutants. We place the results in context by conducting a systematic review and meta-analysis of the published reports on the association between NO_2_ and lung function in children. Finally, we combine results from the meta-analysis with data from CHASE to quantify the impact of changes in air pollution on the prevalence of abnormal lung function in a childhood population.

## Methods

### CHASE study

#### Study population

Full details of CHASE design have been reported elsewhere [12]. Ethical approval was obtained from the Multicentre Research Ethics Committee (Wales). The study was carried out in accordance with the principles expressed in the Declaration of Helsinki. Informed written consent was obtained from parents or guardians. The main study was based in a sample of 200 primary schools in London, Birmingham and Leicester providing balanced numbers of children of South Asian origin (including Indians, Pakistanis, and Bangladeshis), black African-Caribbean origin (including black Africans and black Caribbeans), and white European origin. The present investigation is based in all 183 London primary schools in the study; Birmingham and Leicester schools were excluded because air pollution data were not available for these. A single survey team including three trained research nurses carried out all survey measurements during school terms between October 2004 and February 2007, making two weekly visits to schools in North-West, North-East or South London in rotation. Participating children provided a blood sample and a saliva sample after an overnight fast, had physical measurements and completed questionnaires. Lung function measurements were made with a single Compact 2 pneumotachograph (Vitalograph Ltd, Buckingham, UK), which measures air flow through a resistive mesh on the Fleisch principle and measures volumes by flow integration. The pneumotachograph was calibrated twice daily using standard 5 litre volumes measured by a precision syringe. Spirometric indices were corrected to BTPS (body temperature and pressure, saturated). Following instruction and a practice attempt, each child performed three forced expiratory manoeuvres in the standing position and without nose-clips, according to the methods recommended by the American Thoracic Society [13]. Forced expiratory volume in one second (FEV_1_) and other indices were automatically recorded for the “best” test as defined by the American Thoracic Society [13], based on the maximum sum of FEV_1_ and FVC. Where the variation between FEV_1_ and FVC was >5%, a fourth manoeuvre was performed.

Parents or guardians living with the child provided information on their home address and postcode and on their occupation(s). Information on parental occupation was coded using the National Statistics Socioeconomic Classification (NS-SEC) [14], as previously described [15], defining managerial-professional, intermediate, routine and economically inactive categories. Based on home postcode, information on the small area Index of Multiple Deprivation (IMD) score [16] was obtained for all study participants. Cotinine was measured in saliva using a gas-liquid chromatography method (detection limit 0.1 ng/ml) [17].

#### Long-term air pollution exposure assessment

Annual average concentrations of air pollutants for all locations in London were estimated at a 20x20m resolution using the KCLurban dispersion model, developed as part of the TRAFFIC project [18]. The model provides concentrations of PM_10_ and PM_2.5_ (aerodynamic diameter less than 10µm and 2.5µm, respectively) total, exhaust (tailpipe emissions) and non-exhaust (brake and tyre wearing and resuspension), coarse fraction (obtained by subtracting total PM_2.5_ from total PM_10_), nitric oxide (NO), nitrogen dioxide (NO_2_), nitric oxides (NO_x_), ozone (O_3_) and total oxidants (O_x_), obtained by adding NO_2_ to O_3_. The KCLurban model is based on the Atmospheric Dispersion Modelling System (ADMS) v.4 and road source model v.2.3, measured hourly meteorological data, empirically derived NO, NO_2_, O_3_ and PM relationships and source emissions from the London Atmospheric Emissions Inventory [18]. For linkage to the air pollution data, the current home address of each participant was geocoded, locating the address to the nearest meter. The air pollution concentration was interpolated from the closest 20x20m point of the dispersion model. The annual estimates for 2005 and 2006 were averaged to provide estimates corresponding to the main years of the CHASE survey. Using the Transport for London Traffic Survey, proximity to roads was quantified by obtaining the distance in meters from the subject’s address to the nearest quartiles for heavy traffic (light goods vehicles and heavier) intensity and by estimating the heavy vehicle km travelled within buffers of 100m radius of the address.

#### Statistical analysis

Linear mixed effect models fitting school as a random effect were used to take into account the natural clustering of children within schools. Lung function metrics were included as dependent variables in the different models without transformation. All analyses were adjusted for age, sex, ethnicity, observer, trunk length, indoor room temperature, and month. Secondary analyses were conducted adjusting the estimates also for potential confounding factors including salivary cotinine levels, IMD score, NS-SEC group, sum of skin folds, fat mass index, and having a pet at home.

### Systematic review of the literature on NO_2_ and lung function in children

A systematic literature search was conducted in Medline to identify eligible studies published between 1990 and 2015 reporting cross-sectional associations between NO_2_ and lung function among children or adolescents. The search string is reported in S1 Appendix. The references were then selected by hand according to the following inclusion criteria:

- Study participants were children or adolescents (age 0–18 years).
- The study reported cross-sectional estimates of the association between outdoor NO_2_ levels and FEV_1_, the most commonly reported lung function parameter in the epidemiological literature [5]. We chose not to evaluate the association between NO_2_ and FEF25-75 because too few studies reported estimates for this association. Results from longitudinal studies reporting changes in growth of lung function over time were excluded.
- The study reported linear regression analyses with either untransformed or natural log (ln)–transformed FEV_1_ as dependent variable and NO_2_ levels as a continuous independent variable.
- Information on NO_2_ exposure was available at the individual level or, in the case of aggregated data, the analysis was based on measurements on at least three communities.
- The reported estimates reflected the effect of long term exposures to NO_2_. For this reason, only studies based on an exposure period of at least one year were included.

Relevant details were extracted from the selected papers. Quantitative estimates and their 95% confidence intervals were standardized to a 10 μg/m^3^ increment of NO_2_. NO_2_ levels reported in ppb were converted to μg/m^3^ using the conversion factor adopted by European Commission (1 ppb = 1.91 μg/m^3^) [19]. Studies using linear models and those using log-linear models were analyzed separately. CHASE results were analyzed using both methods and combined with both types of studies. Only one effect estimate per study was included in the meta-analysis. When possible, individual rather than aggregate exposure assessment was used. When estimates based on different periods of exposure were available, the one based on the longer period was used. If periods were of the same length, the one closer to the time of lung function measurements was chosen. When different estimates were based on overlapping data from the same study, the estimate based on the larger sample was chosen. When only results from subgroup analyses (for example gender-specific results) were available in a study, we calculated a pooled estimate for all groups combined and included it in our meta-analysis. One included paper reported a pooled estimate of five studies included in the ESCAPE project [6]. The reported estimates from the five single studies participating in the ESCAPE project were used in the present analysis [6].

We calculated summary effects estimates using random effects models [20], and heterogeneity using the I^2^ statistic [21]. Publication bias was evaluated examining the funnel plots [22] and through two formal tests [23,24].

We carried out sensitivity analyses to evaluate the robustness of our estimates. Analyses were replicated excluding the studies pooled in the ESCAPE project. We also conducted the analysis stratifying by method of exposure assessment used (land use regression and dispersion models versus fixed monitoring stations) and asthma status. The ESCAPE project reported only the pooled estimate of effects stratified by asthma status [6]. For this reason, the pooled estimate rather than the estimates of the single studies included in the ESCAPE project was used for this specific analysis. The study of Urman and colleagues reported results stratified by asthma status only for NO_x_ but not for NO_2_ [25]. For this reason we used a scaling factor of 0.44 to estimate the effects of NO_2_, as previously proposed by Anderson and colleagues [26]. We calculated the combined effect for each stratum and evaluated the difference between strata-specific estimates. The null hypothesis that the difference between the estimates from the two strata equals 0 was tested (with Z-score), and the corresponding p-value is reported here.

### Assessing the health impact of estimated air pollution effects on lung function

A change in the mean values of lung function in the population is not straightforward to interpret in a public health perspective. Indeed, a small reduction in mean physiological measures such as lung function at the population level can translate in a substantial increase in the proportion of subjects with abnormally low lung function [27,28]. For this reason, we evaluated the health impact of traffic-related pollution calculating the proportion of children with abnormal lung function (FEV1<80% of predicted value) expected under different scenarios of NO_2_ exposure, as previously reported [28]. We first calculated the prevalence of children with abnormal lung function in CHASE under the actual levels of exposure to NO_2_ (40 μg/m^3^). Then we shifted the distribution of FEV1 in the population, assuming a 10 μg/m^3^ increase in the levels of NO_2_ and applying the corresponding effect on FEV1 as estimated by the log-linear model in our meta-analysis. Finally, we calculated the proportion of subjects that would have abnormal values of FEV_1_ under this second scenario, and we compared the two proportions.

Analyses were performed with the software Stata 12 (StataCorp, College Station, TX, USA). Statistical significance was defined as p < 0.05 for all analyses.

## Results

### CHASE study

Among 7806 London children invited to participate, 4884 (63%) participated in the study, did not change address during the study period and had valid lung function measurements and complete data on covariates. Participants and non-participants were similar in age, but girls and South Asian ethnicity had higher prevalences among participants than non-participants (by 2% and 3% respectively).

The participants included similar numbers of boys and girls (2396 and 2488 respectively); their mean age was 9.9 (SD 0.4) years (Table 1). The study included similar numbers of children of white European, South Asian, black African-Caribbean and other ethnic origins. Prevalence of reported asthma was 10% in both sexes. About 50% of children had levels of cotinine above 0.1 ng/ml, suggesting a degree of exposure to passive smoking.

**Table 1:** Summary statistics by gender in CHASE study. Values are numbers (percentages) of participants unless stated otherwise.

	Males (n = 2396)	Females (n = 2488)
Mean (SD) age	9.9 (0.4)	9.9 (0.4)
Mean (SD) FVC (ml)	2143 (430)	1995 (402)
Mean (SD) FEV_1_ (ml)	1806 (352)	1728 (342)
Ethnicity		
White European	604 (25.2)	568 (22.8)
Black African-Caribbean	636 (26.5)	717 (28.8)
South Asian	589 (24.6)	617 (24.8)
Asian other	149 (6.2)	176 (7.1)
Other ethnicity	418 (17.5)	410 (16.5)
Reported asthma in the last two years	255 (10.6)	257 (10.3)
Exposure to passive smoking (salivary cotinine >0.1 ng/ml)	1049 (43.8)	1159 (46.6)
Mean (SD) Fat Mass Index (Kg/m^5^)	1.96 (0.95)	2.15 (0.98)
Mean (SD) Sum of skin folds (mm)	42.8 (24.1)	51.0 (24.3)
Mean (SD) Index of Multiple deprivation	30.5 (12.6)	30.7 (12.5)
Having a pet at home	792 (34.2)	821 (34.1)

Variations in exposure to the different pollutants in the study participants are reported in Table 2. Substantial correlations were observed among the estimated concentrations of the different pollutants (S1 Table). As expected, nitrogen oxides and PM concentrations were all strongly positively correlated with each other and inversely correlated with ozone levels.

**Table 2:** Distribution summary of pollutants at the home address of participants and metrics of traffic proximity in CHASE study. Results based on data averaged over 2005 and 2006.

Pollutant	Mean	5^th^ percentile	25^th^ percentile	50^th^ percentile	75^th^ percentile	95^th^ percentile	IQR
NO_2_ (μg/m^3^)	39.7	33.9	37.1	38.9	42.0	47.2	4.9
NO (μg/m^3^)	28.0	19.7	23.4	26.4	31.1	40.4	7.7
NO_x_ (μg/m^3^)	67.7	53.5	60.5	65.5	73.1	87.7	12.6
O_3_ (μg/m^3^)	37.4	33.4	35.9	37.7	38.8	40.9	2.9
Oxidants (μg/m^3^)	77.1	74.8	75.9	76.6	77.9	80.6	2.0
PM_10_ (μg/m^3^)	24.8	23.3	24.1	24.6	25.3	26.8	1.2
PM_10_ exhaust (μg/m^3^)	0.8	0.5	0.7	0.8	0.9	1.3	0.2
PM_10_ non exhaust (μg/m^3^)	2.5	1.7	2.1	2.4	2.7	3.7	0.7
PM_2.5_ (μg/m^3^)	16.0	15.2	15.6	15.9	16.3	17.0	0.7
PM_2.5_ exhaust (μg/m^3^)	0.7	0.5	0.6	0.7	0.8	1.1	0.2
PM_2.5_ non exhaust (μg/m^3^)	0.7	0.5	0.6	0.7	0.8	1.0	0.2
Pm coarse (μg/m^3^)	8.8	8.1	8.5	8.7	9.0	9.7	0.5
Distance of home from highly trafficked roads (meters)	223.4	23.9	92.7	182.9	313.4	559.6	220.8
Vehicle km driven per year within 100m from home	91680	0	0	0	127148	369509	127148
Distance of school from highly trafficked roads (meters)	227.36	40.51	105.06	193.22	311.17	516.20	206.11
Vehicle km driven per year within 100m from school	66813	0	0	0	86823	273970	86823

IQR: inter quartile range

The estimated associations between residential exposure to different pollutants and lung function in CHASE are presented in Table 3. Levels of pollutants (except ozone) showed inverse but non-significant associations with both FEV1 and FVC. Further adjustment for salivary cotinine levels, IMD score, NS-SEC group, sum of skin folds, fat mass index, and having a pet at home did not appreciably change the results (confounder model in Table 3). The results were not changed when pollutant exposure estimates were based on the individual year of lung function measurement, rather than the 2005–6 period. No consistent pattern of association was observed for forced expiratory flows (S2 and S3 Tables). Lung function indices showed no consistent associations with different metrics of traffic intensity (S4 Table)

**Table 3:** Associations between concentration of pollutants and lung function. Absolute differences in lung function for one interquartile range (IQR) increase in the levels of the pollutant. Results expressed in ml.

			Basic model		Confounder model	
	IQR (μg/m^3^)	lung function	Effect of 1 IQR increase in the levels of the pollutant (95% CIs)	p-value	Effect of 1 IQR increase in the levels of the pollutant (95% CIs)	p-value
NO_2_	4.9	FVC	-12 (-25 to 2)	0.08	-9 (-24 to 6)	0.24
		FEV_1_	-5 (-16 to 7)	0.41	-5 (-18 to 8)	0.47
NO	7.7	FVC	-8 (-20 to 4)	0.19	-5 (-18 to 9)	0.49
		FEV_1_	-3 (-13 to 7)	0.57	-2 (-14 to 9)	0.67
NO_x_	12.6	FVC	-9 (-22 to 3)	0.15	-6 (-20 to 8)	0.39
		FEV_1_	-4 (-14 to 7)	0.51	-3 (-15 to 9)	0.60
O_3_	2.9	FVC	14 (-2 to 30)	0.08	10 (-7 to 28)	0.24
		FEV_1_	6 (-7 to 20)	0.37	5 (-9 to 20)	0.48
Oxidants (NO_2_ + O_3_)	2.0	FVC	-9 (-19 to 2)	0.12	-7 (-19 to 5)	0.27
		FEV_1_	-3 (-12 to 6)	0.49	-4 (-14 to 7)	0.49
PM_10_	1.2	FVC	-8 (-20 to 5)	0.24	-5 (-19 to 8)	0.44
		FEV_1_	-3 (-14 to 7)	0.55	-4 (-16 to 8)	0.53
PM_10_ Exhaust	0.2	FVC	-5 (-16 to 6)	0.34	-3 (-16 to 9)	0.59
		FEV_1_	-2 (-11 to 8)	0.70	-2 (-13 to 8)	0.69
PM_10_ Non-exhaust	0.7	FVC	-4 (-13 to 6)	0.46	-2 (-13 to 8)	0.70
		FEV_1_	-1 (-9 to 7)	0.84	-1 (-10 to 8)	0.80
PM_2.5_	0.7	FVC	-11 (-25 to 4)	0.15	-8 (-24 to 8)	0.32
		FEV_1_	-6 (-18 to 6)	0.35	-7 (-20 to 7)	0.35
PM_2.5_ Exhaust	0.2	FVC	-5 (-16 to 6)	0.34	-3 (-16 to 9)	0.59
		FEV_1_	-2 (-11 to 8)	0.70	-2 (-13 to 8)	0.69
PM_2.5_Non-exhaust	0.2	FVC	-3 (-13 to 6)	0.49	-2 (-12 to 8)	0.72
		FEV_1_	-1 (-9 to 7)	0.79	-1 (-10 to 7)	0.74
PM coarse	0.5	FVC	-5 (-16 to 6)	0.37	-3 (-15 to 8)	0.60
		FEV_1_	-1 (-10 to 8)	0.79	-2 (-11 to 8)	0.76

Basic model is adjusted for month, trunk length, ethnic subgroup, observer, sex, age, indoor room temperature and school (as random effect). Confounder model adjusted for all the variables included in the basic model plus cotinine, IMD score, NS-SEC group, sum of skin folds, fat mass index, and having a pet at home.

### Systematic review

**Fig 1:**
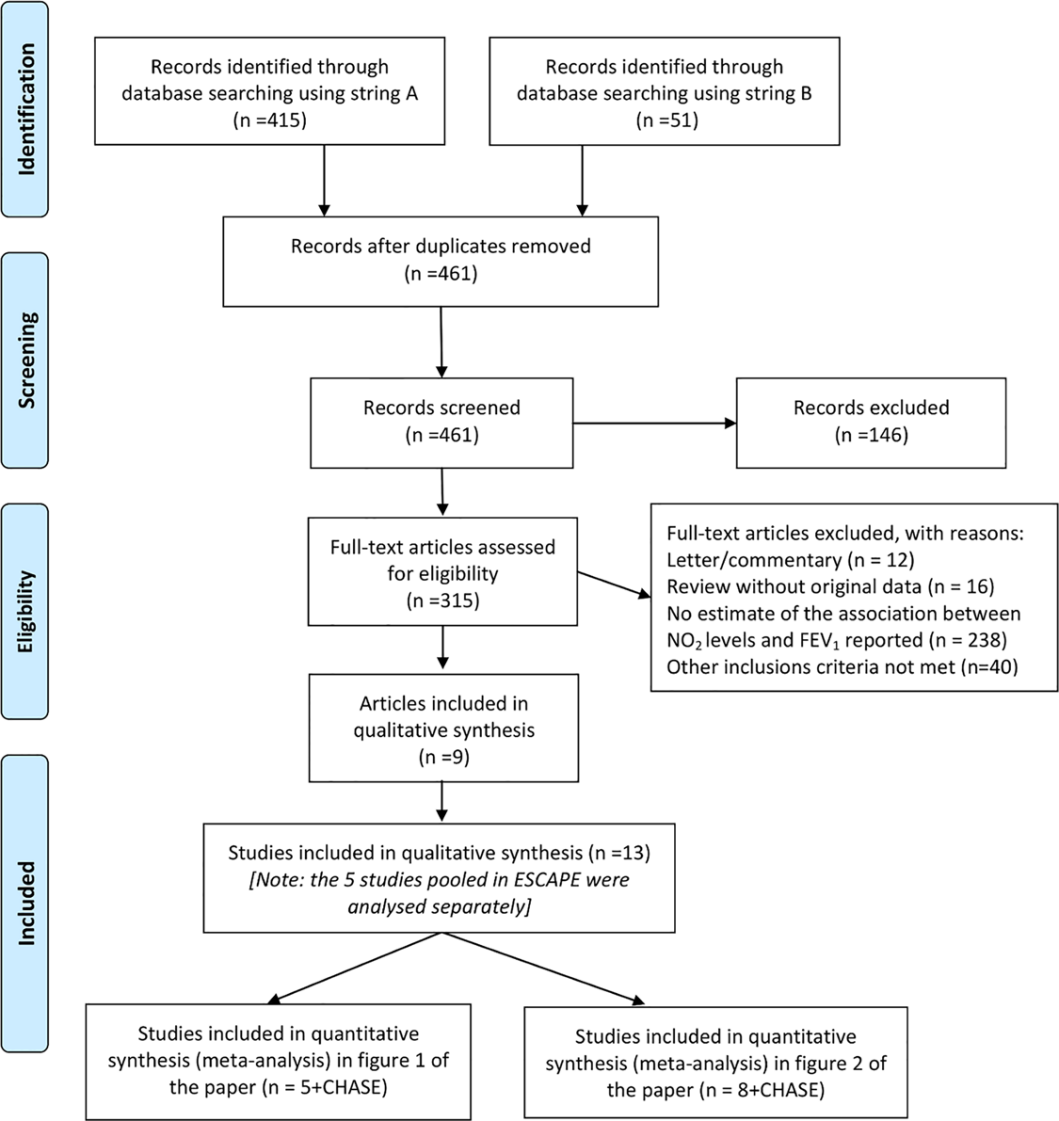
PRISMA Flow-Diagram of the systematic review.

**Fig 2:**
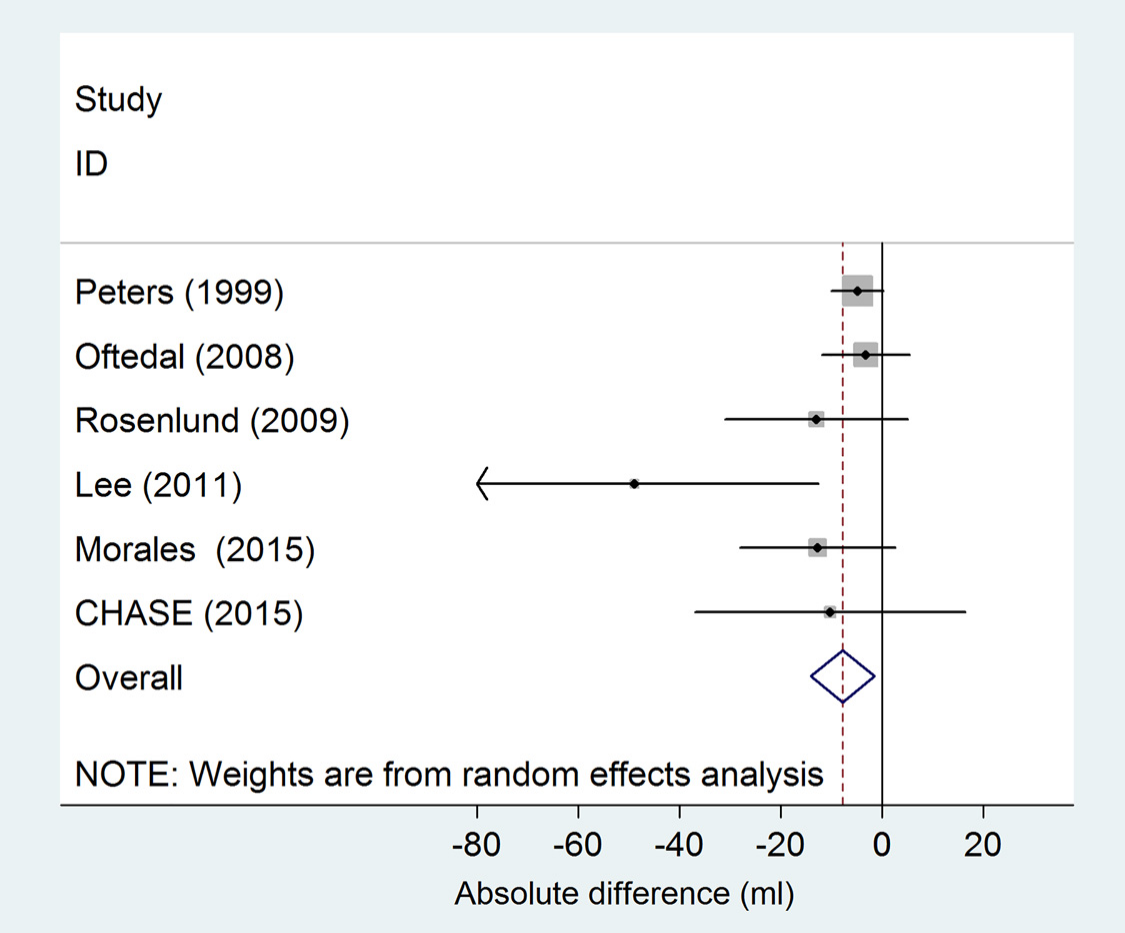
Random effects meta-analysis of the association between concentration of NO_2_ and FEV_1_. Linear models. Results expressed as absolute differences in FEV_1_ for a 10 μg/m^3^ increase in the levels of NO_2_.

**Fig 3:**
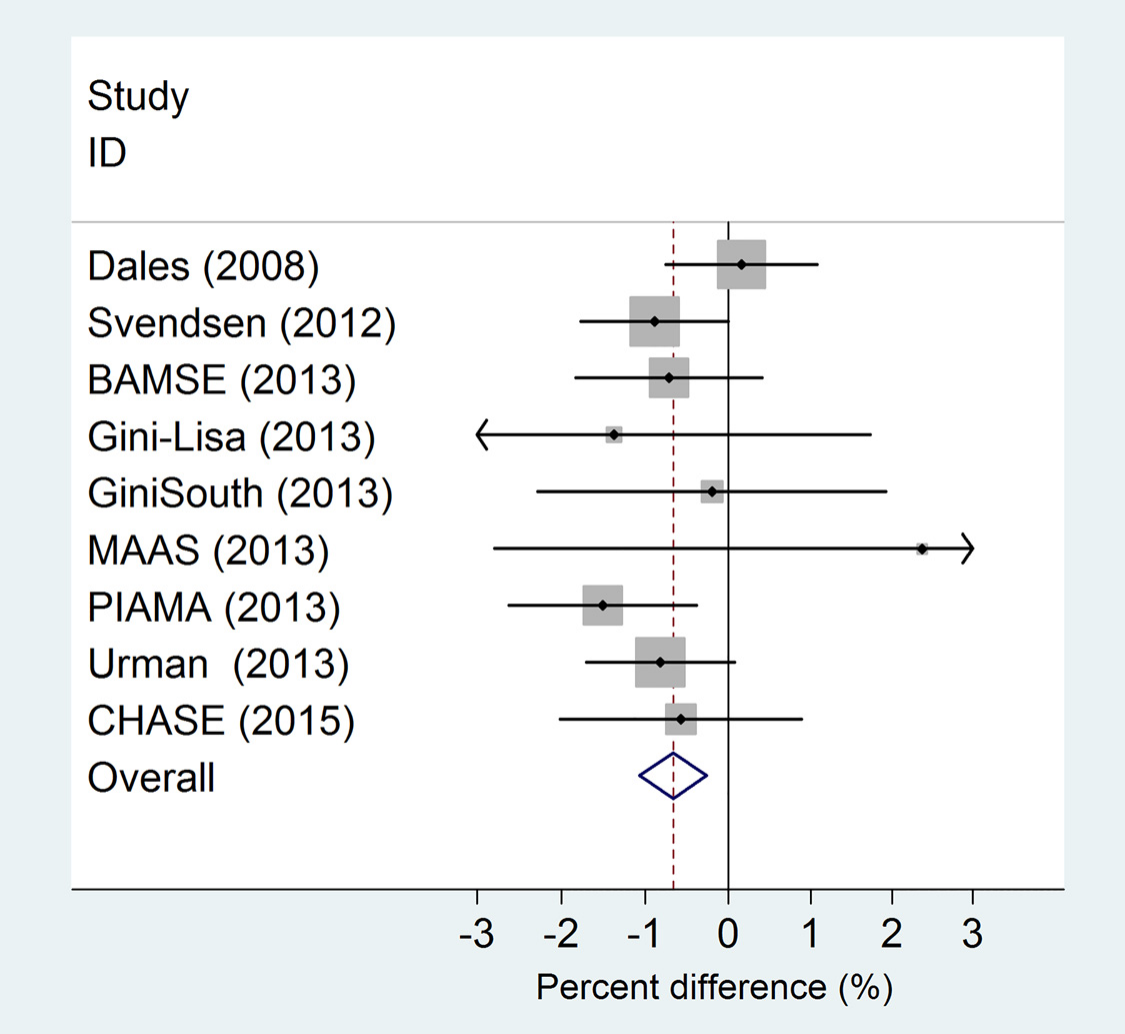
Random effects Meta-Analysis of the association between concentration of NO_2_ and FEV_1_. Log-linear models. Results expressed as percent differences in FEV_1_ for a 10 μg/m^3^ increase in the levels of NO_2_.

The search yielded 461 papers (Fig 1). Among these, 13 studies met our selection criteria (Tables 4 and 5) [6,8,9,25,29–33]. Eight studies were from Europe, 1 from Asia and 4 from North America. The ages of subjects ranged from 4 to 16 years but the majority (9 studies) included children between the ages of 8 and 12 years. The methods of exposure assessment were study-specific monitors (2), land use regression (10), and dispersion models (1). Linear models were used in 5 studies and CHASE (Table 4, Fig 2), while 8 studies and CHASE used log-linear models (Table 5, Fig 3). All of the linear model studies showed inverse associations of which two were statistically significant. In a random effects meta-analysis, a significant effect of NO_2_ on lung function was observed (Fig 2). Among the studies using linear models, a 10 μg/m^3^ increase in NO_2_ was associated with a slightly lower FEV_1_ (Effect Size: -8 ml, 95% CI: -14 to -1 ml; p = 0.016) (Fig 2). There was moderate heterogeneity (I^2^ = 32%), principally driven by the study of Lee and colleagues [32]. Among the 9 studies using log-linear models, 8 showed inverse associations of which only one was statistically significant. Heterogeneity of the estimates of the effect of NO_2_ on FEV_1_ was very small (I^2^<1%). In a random effects meta-analysis, a 10 μg/m^3^ increase in NO_2_ was associated with a small decrease in FEV_1_ (Effect Size: -0.7%, 95% CI: -1.1% to -0.3%; p = 0.001) (Fig 3). In all the analyses, funnel plots were symmetrical and neither the Begg’s nor Egger’s tests suggested small study bias.

**Table 4:** Published studies reporting the effect of NO_2_ on absolute differences in FEV_1_ in children. Results expressed for a 10 μg/m^3^ increase in the levels of NO_2_.

Author	n. subjects	Age	Mean NO_2_ Level (expressed in μg/m3)	Exposure assessment	Exposure period	Absolute difference in FEV_1_ (ml) per 10 μg/m^3^ increase in NO_2_ level
Peters 1999 [29]	2781	9–16	42	fixed monitoring stations	5 years	-5 (-10 to 0)
Oftedal 2008 [30]	2307	9–11	29	Dispersion model	10 years	-3 (-12 to -5)
Rosenlund 2009 [8]	1195	9–14	45	LUR	1 year	-13 (-31 to 5)
Lee 2011 [32]	3957	12–13	13	fixed monitoring stations	1 year	-49 (-85 to 13)
Morales 2015 [33]	567	4–5	29	LUR	1 year	-13 (-28 to 3)
CHASE 2015	4932	9–10	40	Dispersion model	2 years	-10 (-37 to 16)

**Table 5:** Published studies on the effect of NO_2_ on percent reduction in lung function in children. Results expressed for a 10 μg/m3 increase in the levels of NO_2_.

Author	n. subjects	Age	Mean NO_2_ Level (expressed in μg/m3)	Exposure assessment	Exposure period	Percentage (%) difference in FEV_1_ (ml) per 10 μg/m^3^ increase in NO_2_ level
Dales 2008 [31]	2328	9–11	26	LUR	1 year	0.2 (-0.7 to 1.1)
Svendsen 2012 [9]	2032	9–10	50	LUR	1 year	-0.9 (-1.8 to 0.0)
Gehring 2013 (BAMSE) [6]	2527	8	14	LUR	1 year	-0.7 (-1.8 to 0.4)
Gehring 2013 (Gini-Lisa) [6]	627	6	22	LUR	1 year	-1.4 (-4.4 to 1.7)
Gehring 2013 (Gini South) [6]	948	6	23	LUR	1 year	-0.2 (-2.2 to 2.0)
Gehring 2013 (MAAS) [6]	581	8	23	LUR	1 year	2.4 (-2.8 to 7.8)
Gehring 2013 (PIAMA) [6]	1036	8	23	LUR	1 year	-1.5 (-2.6 to -0.4)
Urman 2013 [25]	1811	10–11	25	LUR	1 year	-0.8 (-1.7 to 0.1)
CHASE 2015	4932	9–10	40	Dispersion model	2 years	-0.6 (-2.0 to 0.9)

Several sensitivity analyses were conducted to further evaluate the robustness of the main results. We conducted the analysis stratifying by exposure assessment method (land use regression and dispersion models versus fixed monitoring stations) to evaluate possible heterogeneity of the results. Among studies using linear models, there was no evidence of heterogeneity between the studies using different methods of exposure assessment (p = 0.66). No such analysis could be conducted among the studies using log-linear models, as only one study was based on fixed monitoring stations. However, the exclusion of this study from the meta-analysis did not change the results appreciably. We also did not find evidence of heterogeneity between the studies that were previously included in the ESCAPE project and the other studies (p = 0.30). When we reran the analysis excluding studies from ESCAPE, the estimates were similar to those from the main analysis (Effect Size: -0.5%, 95% CI: -0.9% to -0.0%). Results did not change substantially when we conducted the analysis separately in subjects with and without a diagnosis of asthma. Among studies using linear models, a 10 μg/m^3^ increase in NO_2_ in subjects without a diagnosis of asthma was associated with an 14 ml lower FEV_1_ (95% CI:-26 to -3 ml; p = 0.01). No association was observed for subjects with asthma (Effect Size: 1, 95% CI: -15 to 17 ml; p = 0.90). A similar pattern was also observed in studies using log-linear models (Among not asthmatics, Effect Size: -0.9%, 95% CI: -1.4% to -0.4%, p value = 0.001; among asthmatics, Effect Size: -0.5%, 95% CI: -1.9% to 1%, p value = 0.51).

### Assessing the health impact of estimated air pollution effects on lung function

We observed that in the CHASE population, 7.0% children had a FEV_1_ less than the 80% of the predicted value. According to the results of the meta-analysis, a 10 μg/m^3^ increase in the levels of NO_2_ would be associated with a 0.7% decrease of FEV_1_ in children. The consequent shift in the distribution of the FEV_1_ values would translate in a 7% increase of the prevalence of children with abnormal lung function, from 4.0% to 12%. In absolute terms, this in turn translates in a difference of 507 children per 100,000.

## Discussion

In the present study, we used data from a large cross-sectional study and from a meta-analysis of published studies to evaluate the association between air pollution and lung function in children. Using CHASE data we found consistent, albeit not statistically significant, inverse associations between levels of primary traffic pollutants and lung function in children. When the results of CHASE were included in a meta-analysis of published studies, we found a statistically significant inverse association between NO_2_ and FEV_1_.

The analysis based on CHASE data has several strengths. This is one of the largest individual studies conducted so far to investigate the long-term effects of air pollution on lung function in children. An individual level exposure assessment was used to evaluate the role of a number of air pollutants and traffic proximity metrics at residential level. Moreover, data on several possible confounders at the individual and contextual level were available. In particular, exposure to passive smoking and socio-economic status were assessed very accurately in CHASE. In addition to information from questionnaire, passive smoking exposure was assessed measuring cotinine levels among the participants. Socio-economic status was thoroughly assessed both at the individual level, using information on the parental socio-economic position, and at the contextual level, using an index of multiple deprivation of the neighbourhood where the participant lived. However, estimates of the association between the different air pollutants and lung function did not change appreciably after this adjustment (Table 2). Information on other possible indoor exposures (namely domestic exposure to molds and use of gas for cooking) and on short-term exposure to air pollutants in the days before the lung function measurements were unfortunately not available in our study. One possible limitation of CHASE is the small variation in the levels of exposure among the participants, translating into a relatively low statistical power to find statistically significant associations. For this reason, it is useful to evaluate the results of CHASE in the context of the published literature. To our knowledge, this is the first meta-analysis on the long term effects of NO_2_ on lung function. Gotschi and colleagues in their 2008 review of ambient air pollution and lung function reported that the methods of published studies on air pollution and lung function were too varied to allow meta-analysis [5]. However, only one of the studies used in the present meta-analysis was published at that time [30]; the remaining studies were all published after 2008 and showed consistency in their study designs, exposure assessments, and measured outcomes. Moreover, all providing cross-sectional estimates of the association between NO_2_ levels and FEV1, using similar statistical approaches.

The outcome was measured in the studies according to standardized methods [13]. Exposure to NO_2_ was evaluated at the individual level in most of the included studies.

It is not possible to exclude that the results of our meta-analysis are due to unmeasured confounding at the level of the individual studies. However we note that, with the exception of the study by Peters and colleagues [29], all the studies included in the meta-analysis provided some adjustment for passive smoking exposure at home and socio-economic status, two important possible confounders of the studied association [1]. Although the response rate in CHASE (and in several other studies included in our systematic review) was only moderate, this appeared to have a limited effect on population selection and is unlikely to have appreciably affected exposure-outcome associations [34]. The lack of effect of adjustment for socio-economic status (both in CHASE and in most studies in the meta-analysis) provides further evidence for the validity of our findings. Exposure misclassification is expected to be present to various degrees in the studies included in the meta-analysis. Such misclassification is expected to be non-differential in respect to the lung function values of the participants, resulting in an attenuation of the association. In our meta-analysis, we did not find evidence of publication bias. In our analyses, however, statistical power to detect small studies bias was admittedly low. On the other hand, we note that most of the studies included in our review did not report statistically significant results. For this reason, publication bias is expected to be unlikely among these studies.

Our results suggest an association of NO_2_ with lung function too small in size to be consistently detected by the individual studies so far conducted. Recent reviews of evidence provide support for an association between NO_2_ and several respiratory health effects [35]. This is also in line with the results of recent meta-analyses reporting an association between exposure to NO_2_ and asthma prevalence in within -community spatial studies [11,26]. Our results contribute to the debate suggesting that levels of NO_2_ can be also associated with subclinical respiratory effects in the population. In this respect, it is noteworthy that in our meta-analysis we found a statistically significant association between NO_2_ and FEV1 when we restricted the analysis to children without a diagnosis of asthma. Moreover, the association between NO_2_ and FEV1 among subjects with asthma tended to be weaker rather than stronger. This suggests that the observed effect is not limited to a subgroup of susceptible individuals, but affects the whole population.

The magnitude of the association between NO_2_ levels and lung function estimated in our meta-analysis is comparable to that reported for environmental tobacco smoke. Cook and colleagues reported a 1.4% reduction in FEV_1_ among children exposed to passive smoking at home [36]. According to the results of our meta-analysis, a 25 μg/m^3^ increase of NO_2_ would be associated with a similar reduction in lung function. As the entire population is exposed to outdoor air pollution, the potential health impact of air pollution on lung function can be substantial. According to our calculations, a 10 μg/m^3^ increase of NO_2_ levels would translate into a 7% increase of the prevalence of children with abnormal lung function. Longitudinal studies following children into adulthood are warranted to evaluate whether these deficits in lung function are compensated by a prolonged growth phase, or whether these individuals maintain a reduced lung function during the whole life [5].

One of the main limitations of our meta-analysis is that we used estimates based on single pollutants models. This made impossible to evaluate the effects of NO_2_ per se. Unfortunately, too few studies reported multi-pollutant models to be included in a meta-analysis. This is due to the fact that levels of exposure to the different pollutants are usually very highly correlated, as was also the case in CHASE, and thus multi-pollutant models produce unstable effect estimates. On the other side, if NO_2_ is considered a proxy of traffic pollution, a single pollutant model can be appropriate. Future integration of the epidemiological evidence with chamber studies and toxicological evidence will help to evaluate whether the observed effect is due to NO_2_ rather than to other traffic pollutants [35].

### Conclusions

Exposure to traffic pollution may cause an overall reduction in lung function. This reduction is expected to increase the prevalence of children with clinically relevant declines in lung function.

## Supporting Information

S1 Appendix Search strings used for the systematic review. (DOCX)

S2 Appendix Flow Diagram of the London arm of the CHASE study used for the TRAFFIC study. (PDF)

S3 Appendix Codebook of the dataset of the TRAFFIC/CHASE study. (DOCX)

S1 Dataset Dataset of the TRAFFIC/CHASE study. (TXT)

S1 PRISMA Checklist PRISMA checklist. (DOC)

S1 STROBE Checklist (DOCX)

S1 Table Correlations between pollutants and traffic proximity metrics based on data averaged over 2005 and 2006 in CHASE study. (DOCX)

S2 Table Association between concentration of different pollutants and lung function measurements in CHASE. (DOCX)

S3 Table Association between concentration of different types of particulate matter and lung function measurements in CHASE. (DOCX)

S4 Table Association between different metrics of traffic proximity and lung function measurements in CHASE. (DOCX)

S5 Table Number of participants with missing data for each variable of interest. (DOCX)

S6 Table Additional information on the studies included in the meta-analysis. (DOCX)

## Abbreviations

NO_2_: Nitrogen Dioxide
CHASE: Child Heart and Health Study in England
FEV_1_: Forced Expiratory Volume in 1 second
TRAFFIC: The Traffic Pollution and Health in London
BTPS: standardized to barometric pressure at sea level, body temperature, saturated with water vapor
FVC: Forced Vital Capacity
NS-SEC: National Statistics Socioeconomic Classification
PM_10_: particle matter less than 10 micrometers in diameter
PM_2.5_: particle matter less than 2.5 micrometers in diameter
NO: nitric oxide
NO_x_: nitric oxides
O_3_: ozone
O_x_: total oxidants
ADMS: Atmospheric Dispersion Modelling System
IMD: Index of Multiple Deprivation
SD: Standard Deviation
LUR: Land Use Regression
