# Supplementary material for: Long-Term Exposure to Primary Traffic Pollutants and Lung Function in Children: Cross-Sectional Study and Meta-Analysis
Source: PLoS One. 2015 Nov 30;10(11):e0142565. doi: 10.1371/journal.pone.0142565 (PMC4664276; doi:10.1371/journal.pone.0142565)
Supplement: S1 Appendix — (DOCX) [file pone.0142565.s001.docx]

S1 Appendix. Search strings used for the systematic review. The second string was used to identify relevant papers published from 2013 to 2015 that still did not have Mesh terms attached at the time of the search (June 2015).

**String A. Filters: period 1990-2013, age 0-18, English language**

("Air Pollution/adverse effects"[Mesh:NoExp] OR "Air Pollution/statistics and numerical data"[Mesh:NoExp] OR "Air Pollutants/adverse effects "[Mesh:NoExp] OR "Air Pollutants/toxicity"[Mesh:NoExp] OR "Vehicle Emissions/adverse effects"[Mesh] OR "Nitrogen Dioxide"[Mesh] OR "Nitric Oxide/toxicity"[Mesh]) AND ("Respiratory Function Tests"[Mesh:NoExp] OR "Spirometry"[Mesh:NoExp] OR "Forced Expiratory Volume"[Mesh] OR "Vital Capacity"[Mesh] OR “Lung Volume Measurements”[Mesh:NoExp] OR “Total Lung Capacity”[Mesh:NoExp] OR “Forced Expiratory Flow Rates”[Mesh:NoExp] OR “Peak Expiratory Flow Rate”[Mesh] OR "Asthma/physiopathology"[Mesh:NoExp]) AND ("Child"[Mesh] OR "Adolescent"[Mesh])

**String B. Filters: period 2013-2015, age 0-18, English language.**

("air pollution " OR "nitrogen dioxide" ) AND ("lung function" OR fev1 OR fvc)
