# Supplementary figures and images for: Long-Term Exposure to Primary Traffic Pollutants and Lung Function in Children: Cross-Sectional Study and Meta-Analysis
Source: PLoS One. 2015 Nov 30;10(11):e0142565. doi: 10.1371/journal.pone.0142565 (PMC4664276; doi:10.1371/journal.pone.0142565)

## Flow Diagram of the London arm of the CHASE study used for the TRAFFIC study

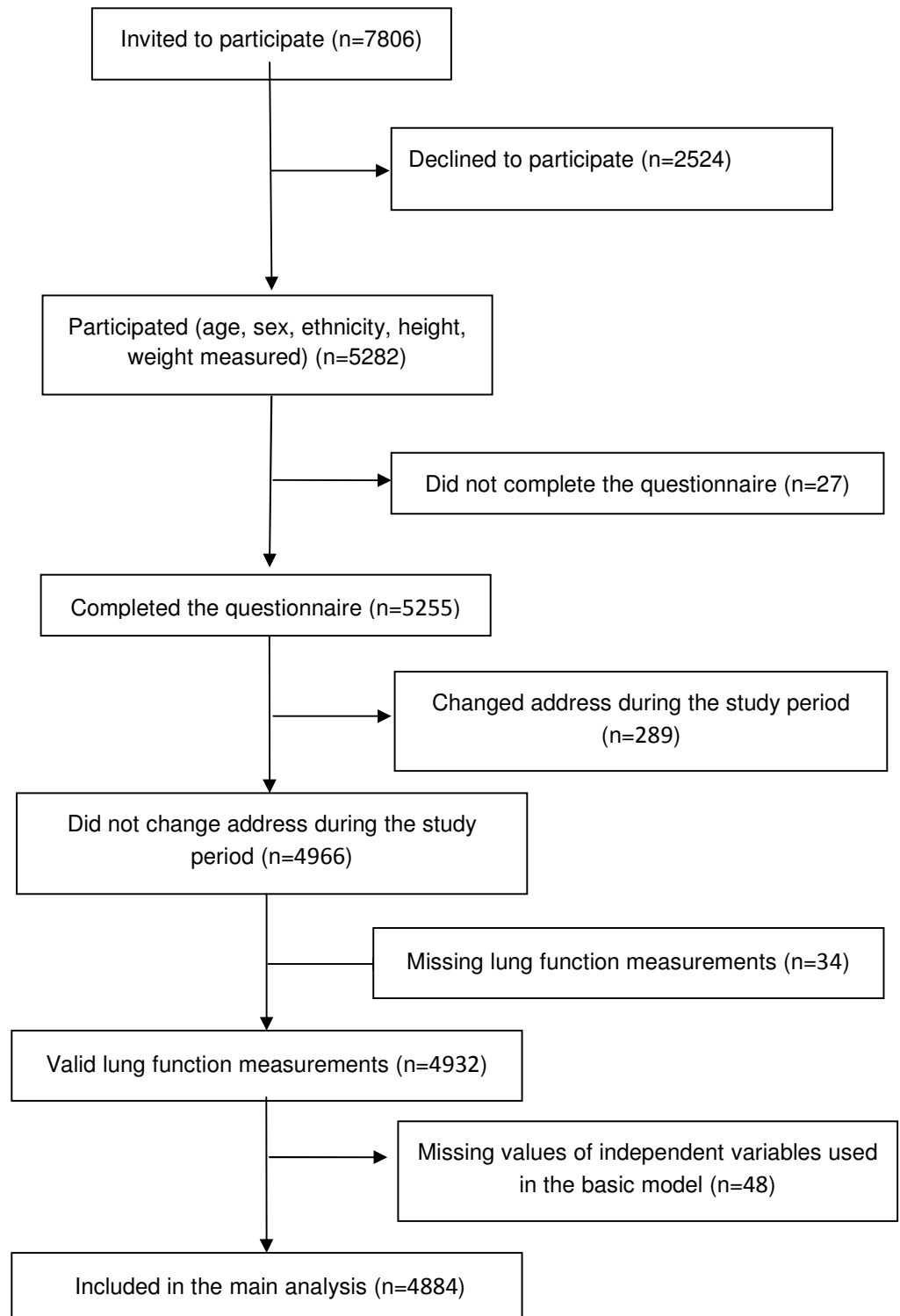

Supplement: S2 Appendix — (PDF) [file pone.0142565.s002.pdf]
