# Supplementary material for: Long-Term Exposure to Primary Traffic Pollutants and Lung Function in Children: Cross-Sectional Study and Meta-Analysis
Source: PLoS One. 2015 Nov 30;10(11):e0142565. doi: 10.1371/journal.pone.0142565 (PMC4664276; doi:10.1371/journal.pone.0142565)
Supplement: S3 Appendix — (DOCX) [file pone.0142565.s003.docx]

**List of variables**

Id: subject’s ID

id_school: School’s ID

no: nitric oxide (NO) levels, expressed in μg/m^3^

no2: nitrogen dioxide (NO2) levels, expressed in μg/m^3^

nox: nitric oxides (NOx) levels, expressed in μg/m^3^

o3: ozone (O3) levels, expressed in μg/m^3^

ox: total oxidants levels (NO_2_ and O_3_) levels, expressed in μg/m^3^

pm10: PM10 levels, expressed in μg/m^3^

pm10exh: exhausted PM10 levels, expressed in μg/m^3^

pm10nonex: non exhausted PM10 levels, expressed in μg/m^3^

pm25: PM2.5 levels, expressed in μg/m^3^

pm25exh: exhausted PM2.5 levels, expressed in μg/m^3^

pm25nonexh: non exhausted PM2.5 levels, expressed in μg/m^3^

pmcoarse: PM coarse fraction levels, expressed in μg/m^3^

fvc: FVC expressed in liters

fev1: FEV1 expressed in liters

fef25: FEF25 expressed in liters/second

fef50: FEF50 expressed in liters/second

fef75: FEF75 expressed in liters/second

month: month when the lung function measurement was performed

trunk: trunk length expressed in cm.

ethnic: ethnic group of the subject (1:White European 2: Black African-Caribbean 3: South Asian 4: Asian other 5: Other ethnicity)

observer: field technician who performed the lung function measurements

sex: 0 male; 1 female

age: age expressed in quartiles

temp_room: indoor room temperature, standardized and expressed as z-value

cotinine: serum cotinine levels, expressed in ng/ml

imd: Index of Multiple Deprivation

nssec: NS-SEC group: 1 Managerial & professional occupations; 2 Intermediate occupations; 3 Routine & manual occupations; 4 Inactive; 5 Unclassified ; 6 missing.

sum_skf: sum of skinfolds expressed in mm

fmi: Fat Mass Index expressed in kg/m^5^

pet: having a pet at home
