# Supplementary material for: Long-Term Exposure to Primary Traffic Pollutants and Lung Function in Children: Cross-Sectional Study and Meta-Analysis
Source: PLoS One. 2015 Nov 30;10(11):e0142565. doi: 10.1371/journal.pone.0142565 (PMC4664276; doi:10.1371/journal.pone.0142565)
Supplement: S1 STROBE Checklist — (DOCX) [file pone.0142565.s006.docx]

STROBE Statement—checklist of items that should be included in reports of observational studies

|  | Item No. | Recommendation | Page  No. | Relevant text from manuscript |
| --- | --- | --- | --- | --- |
| **Title and abstract** | 1 | (*a*) Indicate the study’s design with a commonly used term in the title or the abstract | 1 | “Cross-sectional study” |
|  |  | (*b*) Provide in the abstract an informative and balanced summary of what was done and what was found | 2 | “We investigated the associations between lung function and long-term exposure to a range of primary traffic-related pollutants and background pollutants in a cross-sectional study of London children. […] In CHASE, there was a general negative association between levels of pollutants and lung function, but none of the associations was statistically significant.” |
| Introduction | | | |  |
| Background/rationale | 2 | Explain the scientific background and rationale for the investigation being reported | 3 | “Ambient concentrations of urban air pollution comprise a variety of particulate and gaseous pollutants emanating from both urban and external sources. Of particular concern in modern cities is pollution from traffic sources, and its possible effects on respiratory health in children. Lung function is an objective marker of respiratory health, as well as a predictor of longer-term cardiorespiratory morbidity and mortality. However, the association between long-term exposure to air pollution and lung function in children is still uncertain.” |
| Objectives | 3 | State specific objectives, including any prespecified hypotheses | 4 | “Here we report on an investigation linking air pollution exposures with lung function in a large cross-sectional study of lung function in 9-10 year-old children living in London, The Child Heart and Health Study in England (CHASE). The primary hypothesis concerns the effects of near-traffic primary pollutants since these have the greatest influence on variations in exposure within the city. “ |
| Methods | | | |  |
| Study design | 4 | Present key elements of study design early in the paper | 5 | *[note of the authors: key elements of the study are presented in the first paragraph of the Methods sections. Please refer to the points 5-10 of this checklist]* |
| Setting | 5 | Describe the setting, locations, and relevant dates, including periods of recruitment, exposure, follow-up, and data collection | 5 | “The main study was based in a sample of 200 primary schools in London, Birmingham and Leicester providing balanced numbers of children of South Asian origin (including Indians, Pakistanis, and Bangladeshis), black African-Caribbean origin (including black Africans and black Caribbeans), and white European origin. The present investigation is based in the 183 London primary schools in the study. A single survey team including three trained research nurses carried out all survey measurements during school terms between October 2004 and February 2007, making two weekly visits to schools in North-West, North-East or South London in rotation.” |
| Participants | 6 | (*a*) *Cohort study*—Give the eligibility criteria, and the sources and methods of selection of participants. Describe methods of follow-up  *Case-control study*—Give the eligibility criteria, and the sources and methods of case ascertainment and control selection. Give the rationale for the choice of cases and controls  *Cross-sectional study*—Give the eligibility criteria, and the sources and methods of selection of participants | 5  5  Reference 12 (general description of the CHASE study) | “Full details of CHASE design have been reported elsewhere [12].” *[note of the authors: Reference 12 is a paper published in PLoS Medicine. It is easily accessible to any interested reader]*  “The present investigation is based in the 183 London primary schools in the study.”  “The school sampling frame included all state primary schools in London, Birmingham, and Leicester with between 15% and 50% pupils of white European origin. Two separate random samples,  each of 100 schools, were taken. The first included schools in  which pupils of South Asian origin comprised 20%–80% of all pupils, stratified by Indian, Pakistani, and Bangladeshi origin. The second included schools in which pupils of black African-Caribbean origin comprised 20%–80% of all pupils, stratified by African and Caribbean origin. All Head Teachers were approached by the Principal Investigator and invited to participate; 140 (70%) agreed. Non-participating schools were replaced by a school from the sampling frame with a similar ethnic mix and  in the same or a neighbouring borough. The combined sample included 183 London (between 2 and 19 schools in each of 20  London boroughs), 14 Birmingham, and 3 Leicester schools. Depending on school size, either one or two classes of children were invited to participate.” |
|  |  | (*b*) *Cohort study*—For matched studies, give matching criteria and number of exposed and unexposed  *Case-control study*—For matched studies, give matching criteria and the number of controls per case |  |  |
| Variables | 7 | Clearly define all outcomes, exposures, predictors, potential confounders, and effect modifiers. Give diagnostic criteria, if applicable | 6 | “Linear mixed effect models fitting school as a random effect were used to take into account the natural clustering of children within schools. Lung function metrics were included as dependent variables in the different models without transformation. All analyses were adjusted for age, sex, ethnicity, observer, trunk length, indoor room temperature, and month. Secondary analyses were conducted adjusting the estimates also for potential confounding factors including salivary cotinine levels, IMD score, NS-SEC group, sum of skin folds, fat mass index.” |
| Data sources/ measurement | 8* | For each variable of interest, give sources of data and details of methods of assessment (measurement). Describe comparability of assessment methods if there is more than one group | 5  6 | “Participating children provided a blood sample and a saliva sample after an overnight fast, had physical measurements and completed questionnaires. Lung function measurements were made with a single Compact 2 pneumotachograph (Vitalograph Ltd, Buckingham, UK), which measures air flow through a resistive mesh on the Fleisch principle and measures volumes by flow integration. The pneumotachograph was calibrated twice daily using standard 5 litre volumes measured by a precision syringe. Spirometric indices were corrected to BTPS. Following instruction and a practice attempt, each child performed three forced expiratory manoeuvres in the standing position and without nose-clips, according to the methods recommended by the American Thoracic Society. Forced expiratory volume in one second (FEV1) and other indices were automatically recorded for the “best” test as defined by the American Thoracic Society, based on the maximum sum of FEV1 and FVC. Where the variation between FEV1 and FVC was >5%, a fourth manoeuvre was performed. Parents or guardians living with the child provided information on their home address and postcode and on their occupation(s), coded using the National Statistics Socioeconomic Classification (NS-SEC). Cotinine was measured in saliva using a gas-liquid chromatography method (detection limit 0.1 ng/ml). “  “Annual concentrations of air pollutants were estimated at a 20x20m resolution using the KCLurban dispersion model, developed as part of the TRAFFIC project [Beevers 2013]. The model provides concentrations of PM10 and PM2.5 (aerodynamic diameter less than 10µm and 2.5µm, respectively) total, exhaust (tailpipe emissions) and non-exhaust (brake and tyre wearing and resuspension), coarse fraction (obtained by subtracting total PM2.5 from total PM10), nitric oxide (NO), nitrogen dioxide (NO2), nitric oxides (NOx), ozone (O3) and total oxidants (Ox), obtained by adding NO2 to O3. The KCLurban model is based on the Atmospheric Dispersion Modelling System (ADMS) v.4 and road source model v.2.3, measured hourly meteorological data, empirically derived NO, NO2, O3 and PM relationships and source emissions from the London Atmospheric Emissions Inventory [Beevers 2013]. For linkage to the lung function data, the home address of each participant was geocoded at 6 digit level and the air pollution concentration was interpolated from the closest 20m2 point of the dispersion model. The annual estimates for 2005 and 2006 were averaged to provide estimates corresponding to the main years of the CHASE survey. Using the Transport for London Traffic Survey, proximity to roads was quantified by obtaining the distance in meters from the subject’s address to the nearest quartiles for heavy traffic (light goods vehicles and heavier) intensity and by estimating the heavy vehicle km travelled within buffers of 100m radius of the address.” |
| Bias | 9 | Describe any efforts to address potential sources of bias | 13 | An individual level exposure assessment was used to evaluate the role of a number of air pollutants and traffic proximity metrics at residential level. Moreover, data on several possible confounders at the individual and contextual level were available. In particular, exposure to passive smoking and socio-economic status were assessed very accurately in CHASE. In addition to information from questionnaire, passive smoking exposure was assessed measuring cotinine levels among the participants. Socio-economic status was thoroughly assessed both at the individual level, using information on the parental socio-economic position, and at the contextual level, using an index of multiple deprivation of the neighbourhood where the participant lived. |
| Study size | 10 | Explain how the study size was arrived at | 10 | “Among the 7806 London children invited to participate, 4932 completed the questionnaire, did not change address during the study period and had valid lung function measurements. After the exclusion of 44 children that had missing values in some of the variables, the main analysis was conducted on 4884 children. “ *[note of the authors: please also refer to the CHASE flow diagram]* |

Continued on next page

| Quantitative variables | 11 | Explain how quantitative variables were handled in the analyses. If applicable, describe which groupings were chosen and why | 6 | “Lung function metrics were included as dependent variables in the different models without transformation.” |
| --- | --- | --- | --- | --- |
| Statistical methods | 12 | (*a*) Describe all statistical methods, including those used to control for confounding | 6 | “Linear mixed effect models fitting school as a random effect were used to take into account the natural clustering of children within schools. Lung function metrics were included as dependent variables in the different models without transformation. All analyses were adjusted for age, sex, ethnicity, observer, trunk length, indoor room temperature, and month. Secondary analyses were conducted adjusting the estimates also for potential confounding factors including salivary cotinine levels, IMD score, NS-SEC group, sum of skin folds, fat mass index.” |
|  |  | (*b*) Describe any methods used to examine subgroups and interactions |  | NA |
|  |  | (*c*) Explain how missing data were addressed |  | NA *[note of the authors: As shown in the CHASE diagram and in table S6, less than 1% of the individuals in the study had missing values for any independent variable included in the main analysis. ]* |
|  |  | (*d*) *Cohort study*—If applicable, explain how loss to follow-up was addressed  *Case-control study*—If applicable, explain how matching of cases and controls was addressed  *Cross-sectional study*—If applicable, describe analytical methods taking account of sampling strategy | 6 | “Linear mixed effect models fitting school as a random effect were used to take into account the natural clustering of children within schools.” |
|  |  | (*e*) Describe any sensitivity analyses | 6 | “Secondary analyses were conducted adjusting the estimates also for potential confounding factors including salivary cotinine levels, IMD score, NS-SEC group, sum of skin folds, fat mass index.” |
| Results | | | | |
| Participants | 13* | (a) Report numbers of individuals at each stage of study—eg numbers potentially eligible, examined for eligibility, confirmed eligible, included in the study, completing follow-up, and analysed |  | Please refer to the CHASE flow diagram |
|  |  | (b) Give reasons for non-participation at each stage |  | Please refer to CHASE flow diagram |
|  |  | (c) Consider use of a flow diagram |  | Please check the CHASE flow diagram |
| Descriptive data | 14* | (a) Give characteristics of study participants (eg demographic, clinical, social) and information on exposures and potential confounders | 10 | “The participants included similar numbers of boys and girls (2418 and 2514 respectively); their mean age was 9.9 (SD 0.4) years (table 1). The study included similar numbers of children of white European, South Asian, black African-Caribbean and other ethnic origins. Prevalence of reported asthma was 10% in both sexes. About 50% of children had levels of cotinine above 0.1 ng/ml, suggesting a degree of exposure to passive smoking.” |
|  |  | (b) Indicate number of participants with missing data for each variable of interest |  | Please refer to Table S6 |
|  |  | (c) *Cohort study*—Summarise follow-up time (eg, average and total amount) |  |  |
| Outcome data | 15* | *Cohort study*—Report numbers of outcome events or summary measures over time |  |  |
|  |  | *Case-control study—*Report numbers in each exposure category, or summary measures of exposure |  |  |
|  |  | *Cross-sectional study—*Report numbers of outcome events or summary measures |  | Please refer to Table 1 |
| Main results | 16 | (*a*) Give unadjusted estimates and, if applicable, confounder-adjusted estimates and their precision (eg, 95% confidence interval). Make clear which confounders were adjusted for and why they were included | 10 | “The estimated associations between residential exposure to different pollutants and lung function in CHASE are presented in Table 2. There was a general negative association between levels of pollutants and lung function, but none of the associations was statistically significant. There was no material difference between primary traffic pollutants and background pollutants. Further adjustment for salivary cotinine levels, IMD score, NS-SEC group, sum of skin folds, and fat mass index did not appreciably change the results (confounder model in table 2). A similar pattern was observed for forced expiratory flows (Supplementary table S4). Lung function indices showed no consistent associations with different metrics of traffic intensity (Supplementary table S5).” |
|  |  | (*b*) Report category boundaries when continuous variables were categorized |  | NA |
|  |  | (*c*) If relevant, consider translating estimates of relative risk into absolute risk for a meaningful time period |  | NA |

Continued on next page

| Other analyses | 17 | Report other analyses done—eg analyses of subgroups and interactions, and sensitivity analyses | 10 | “Further adjustment for salivary cotinine levels, IMD score, NS-SEC group, sum of skin folds, and fat mass index did not appreciably change the results (confounder model in table 2).” |
| --- | --- | --- | --- | --- |
| Discussion | | | | |
| Key results | 18 | Summarise key results with reference to study objectives | 13 | “In the present study, we used data from a large cross-sectional study and from a meta-analysis of published studies to evaluate the association between air pollution and lung function in children. Using CHASE data we found consistent, albeit not statistically significant, inverse associations between levels of different air pollutants and lung function in children. There was no material difference between primary traffic pollutants (such as NOx and PM2.5 exhaust) and regional/urban background pollutants (such as PM2.5). When the results of CHASE were included in a meta-analysis of published studies, we found a statistically significant inverse association between NO2 and FEV1.” |
| Limitations | 19 | Discuss limitations of the study, taking into account sources of potential bias or imprecision. Discuss both direction and magnitude of any potential bias |  | “One possible limitation of CHASE is the small variation in the levels of exposure among the participants, translating into a relatively low statistical power to find statistically significant associations. For this reason, it is useful to evaluate the results of CHASE in the context of the published literature.” *[note of the authors: a detailed discussion on the limitation of the results of the meta-analysis is provided at page 14, but is not relevant for the present checklist, which is specific for CHASE study]* |
| Interpretation | 20 | Give a cautious overall interpretation of results considering objectives, limitations, multiplicity of analyses, results from similar studies, and other relevant evidence | 14 | *[Note of the authors: we conducted a systematic review of the relevant literature and a meta-analysis. Our interpretation is based on both the results of CHASE and the meta-analysis. Please also check the accompanying PRISMA checklist].*  “Our results suggest an association of NO2 with lung function too small in size to be consistently detected by the individual studies so far conducted. Recent reviews of evidence provide support for an association between NO2 and several respiratory health effects . This is also in line with the results of recent meta-analyses reporting an association between exposure to NO2 and asthma prevalence in within -community spatial studies.” |
| Generalisability | 21 | Discuss the generalisability (external validity) of the study results |  | NA *[note of the authors: the results of the studies are based largely on the meta-analysis, not only on the results of CHASE. Issues of generalizability are discussed for the results of the meta-analysis, not for the results of CHASE only]* |
| Other information | |  | | |
| Funding | 22 | Give the source of funding and the role of the funders for the present study and, if applicable, for the original study on which the present article is based | 16 | “The TRAFFIC study was supported by a cross-Research Council Environmental Exposure and Health Initiative (EEHI) grant (grant reference NE/I008039/1) with funds from the Natural Environment Research Council (NERC), the Medical Research Council (MRC), and the Department of Health (DoH). Data collection in the CHASE Study was supported by grant funding from the Wellcome Trust (grant reference 068362/Z/02/Z). The funders had no role in study design, data collection and analysis, decision to publish, or preparation of the manuscript.” |

*Give information separately for cases and controls in case-control studies and, if applicable, for exposed and unexposed groups in cohort and cross-sectional studies.

**Note:** An Explanation and Elaboration article discusses each checklist item and gives methodological background and published examples of transparent reporting. The STROBE checklist is best used in conjunction with this article (freely available on the Web sites of PLoS Medicine at http://www.plosmedicine.org/, Annals of Internal Medicine at http://www.annals.org/, and Epidemiology at http://www.epidem.com/). Information on the STROBE Initiative is available at www.strobe-statement.org.
