# Supplementary material for: Long-Term Exposure to Primary Traffic Pollutants and Lung Function in Children: Cross-Sectional Study and Meta-Analysis
Source: PLoS One. 2015 Nov 30;10(11):e0142565. doi: 10.1371/journal.pone.0142565 (PMC4664276; doi:10.1371/journal.pone.0142565)
Supplement: S1 Table — (DOCX) [file pone.0142565.s007.docx]

S1 Table. Correlations between pollutants and traffic proximity metrics based on data averaged over 2005 and 2006 in CHASE study.

| Pollutant | NO_2_ | NO | NO_x_ | O_3_ | Oxidants | PM_10_ | PM_10_ exhaust | PM_10_ non exhaust | PM_2.5_ | PM_2.5_ exhaust | PM_2.5_  non exhaust | PM coarse | Distance of home from the road | Km driven around home (100 m) | Distance of school from the road | Km driven around school (100 m) |
| --- | --- | --- | --- | --- | --- | --- | --- | --- | --- | --- | --- | --- | --- | --- | --- | --- |
| NO_2_ | 1 |  |  |  |  |  |  |  |  |  |  |  |  |  |  |  |
| NO | 0.98 | 1 |  |  |  |  |  |  |  |  |  |  |  |  |  |  |
| NO_x_ | 0.99 | 1 | 1 |  |  |  |  |  |  |  |  |  |  |  |  |  |
| O_3_ | -0.98 | -0.94 | -0.96 | 1 |  |  |  |  |  |  |  |  |  |  |  |  |
| Oxidants | 0.97 | 0.97 | 0.97 | -0.90 | 1 |  |  |  |  |  |  |  |  |  |  |  |
| PM_10_ | 0.96 | 0.95 | 0.96 | -0.93 | 0.95 | 1 |  |  |  |  |  |  |  |  |  |  |
| PM_10_ exhaust | 0.95 | 0.95 | 0.95 | -0.90 | 0.96 | 0.98 | 1 |  |  |  |  |  |  |  |  |  |
| PM_10_ non exhaust | 0.90 | 0.92 | 0.92 | -0.84 | 0.93 | 0.97 | 0.97 | 1 |  |  |  |  |  |  |  |  |
| PM_2.5_ | 0.96 | 0.93 | 0.95 | -0.95 | 0.92 | 0.99 | 0.96 | 0.91 | 1 |  |  |  |  |  |  |  |
| PM_2.5_ exhaust | 0.95 | 0.95 | 0.95 | -0.9 | 0.96 | 0.98 | 1 | 0.97 | 0.96 | 1 |  |  |  |  |  |  |
| PM_2.5_ non exhaust | 0.89 | 0.91 | 0.91 | -0.83 | 0.92 | 0.96 | 0.96 | 1 | 0.90 | 0.96 | 1 |  |  |  |  |  |
| PM coarse | 0.93 | 0.93 | 0.93 | -0.87 | 0.94 | 0.98 | 0.98 | 1 | 0.93 | 0.98 | 0.99 | 1 |  |  |  |  |
| Distance of home from the road | -0.41 | -0.41 | -0.41 | 0.37 | -0.43 | -0.44 | -0.43 | -0.47 | -0.41 | -0.43 | -0.47 | -0.46 | 1 |  |  |  |
| Km driven around home (100 m) | 0.51 | 0.56 | 0.54 | -0.44 | 0.57 | 0.58 | 0.61 | 0.66 | 0.51 | 0.61 | 0.66 | 0.64 | -0.40 | 1 |  |  |
| Distance of school from the road | -0.25 | -0.22 | -0.24 | 0.26 | -0.23 | -0.25 | -0.24 | -0.23 | -0.26 | -0.24 | -0.23 | -0.24 | 0.24 | -0.10 | 1 |  |
| Km driven around school (100 m) | 0.20 | 0.19 | 0.19 | -0.19 | 0.19 | 0.16 | 0.17 | 0.16 | 0.16 | 0.17 | 0.17 | 0.16 | -0.11 | 0.08 | -0.39 | 1 |
