# Supplementary material for: Long-Term Exposure to Primary Traffic Pollutants and Lung Function in Children: Cross-Sectional Study and Meta-Analysis
Source: PLoS One. 2015 Nov 30;10(11):e0142565. doi: 10.1371/journal.pone.0142565 (PMC4664276; doi:10.1371/journal.pone.0142565)
Supplement: S2 Table — (DOCX) [file pone.0142565.s008.docx]

S2 TABLE. Association between concentration of different pollutants and lung function measurements in CHASE. Absolute differences in lung function for one interquartile range (IQR) increase in the levels of the pollutant. Basic model adjusted for month, trunk length, ethnic subgroup, observer, sex, age, indoor room temperature and school (as random effect). Confounder model adjusted for all the variables included in the basic model plus cotinine, imd score, nssec group, sum of skin folds, fat mass index, and having a pet at home.

|  |  | Basic model |  | Confounder model |  |
| --- | --- | --- | --- | --- | --- |
|  | lung function | Effect of 1 IQR increase in the levels of the pollutant (95% CIs) | p-value | Effect of 1 IQR increase in the levels of the pollutant (95% CIs) | p-value |
| NO_2_ | FEF_25_ | -5 (-40 to 29) | 0.75 | -2 (-33 to 30) | 0.92 |
|  | FEF_50_ | 0 (-26 to 25) | 0.98 | -3 (-26 to 22) | 0.87 |
|  | FEF_75_ | 4 (-12 to 19) | 0.62 | 2 (-13 to 16) | 0.82 |
| NO | FEF_25_ | -4 (-35 to 26) | 0.78 | 0 (-30 to 29) | 0.98 |
|  | FEF_50_ | 2 (-20 to 25) | 0.84 | 1 (-21 to 23) | 0.91 |
|  | FEF_75_ | 6 (-8 to 20) | 0.38 | 4 (-11 to 19) | 0.62 |
| NO_x_ | FEF_25_ | -5 (-37 to 27) | 0.77 | -1 (-31 to 29) | 0.96 |
|  | FEF_50_ | 1 (-22 to 25) | 0.91 | 0 (-23 to 23) | 0.99 |
|  | FEF_75_ | 5 (-9 to 20) | 0.46 | 3 (-11 to 17) | 0.65 |
| O_3_ | FEF_25_ | 11 (-29 to 51) | 0.59 | 7 (-28 to 41) | 0.71 |
|  | FEF_50_ | 4 (-25 to 34) | 0.77 | 6 (-20 to 32) | 0.65 |
|  | FEF_75_ | -2 (-19 to 16) | 0.83 | 0 (-15 to 16) | 0.96 |
| Oxidants | FEF_25_ | -1 (-29 to 26) | 0.93 | 3 (-26 to 31) | 0.87 |
|  | FEF_50_ | 2 (-18 to 23) | 0.82 | 1 (-20 to 23) | 0.90 |
|  | FEF_75_ | 5 (-8 to 17) | 0.46 | 3 (-10 to 16) | 0.64 |
