# Supplementary material for: Long-Term Exposure to Primary Traffic Pollutants and Lung Function in Children: Cross-Sectional Study and Meta-Analysis
Source: PLoS One. 2015 Nov 30;10(11):e0142565. doi: 10.1371/journal.pone.0142565 (PMC4664276; doi:10.1371/journal.pone.0142565)
Supplement: S4 Table — (DOCX) [file pone.0142565.s010.docx]

S4 Table. Association between different metrics of traffic proximity and lung function measurements in CHASE. Absolute differences in lung function for one interquartile range (IQR) increase in the levels of the traffic proximity metrics. Basic model adjusted for month, trunk length, ethnic subgroup, observer, sex, age, indoor room temperature and school (as random effect). Confounder model adjusted for all the variables included in the basic model plus cotinine, IMD score, NS-SEC group, sum of skin folds, fat mass index.

|  |  | Basic model |  | Confounder model |  |
| --- | --- | --- | --- | --- | --- |
|  | lung function | Effect of 1 IQR increase in the metric (95% CIs) | p-value | Effect of 1 IQR increase in the metric (95% CIs) | p-value |
| Distance of home from highly trafficked roads (meters) | FVC | 6 (-6 to 18) | 0.34 | 6 (-7 to 18) | 0.37 |
|  | FEV_1_ | 5 (-5 to 16) | 0.32 | 5 (-6 to 15) | 0.37 |
|  | FEF_25_ | 25 (-1 to 56) | 0.12 | 21 (-11 to 53) | 0.19 |
|  | FEF_50_ | 13 (-10 to 36) | 0.29 | 11 (-13 to 36) | 0.36 |
|  | FEF_75_ | -5 (-19 to 10) | 0.55 | -5 (-20 to 10) | 0.49 |
| Vehicle km driven per year within 100m from home | FVC | 1 (-5 to 8) | 0.70 | 1 (-5 to 8) | 0.69 |
|  | FEV_1_ | 1 (-4 to 10) | 0.69 | 1 (-5 to 6) | 0.80 |
|  | FEF_25_ | -4 (-20 to 13) | 0.67 | -5 (-21 to 12) | 0.59 |
|  | FEF_50_ | -2 (-15 to 11) | 0.77 | -2 (-15 to 11) | 0.73 |
|  | FEF_75_ | 6 (-2 to 13) | 0.14 | 5 (-3 to 13) | 0.18 |
| Distance of school from highly trafficked roads (meters) | FVC | 0 (-25 to 26) | 0.98 | -0.001 (-27 to 25) | 0.93 |
|  | FEV_1_ | -12 (-34 to 10) | 0.26 | -0.012 (-34 to 9) | 0.25 |
|  | FEF_25_ | -21 (-84 to 42) | 0.51 | -21 (-85 to 43) | 0.52 |
|  | FEF_50_ | -27 (-69 to 15) | 0.21 | -0.024 (-0.067 to 0.019) | 0.28 |
|  | FEF_75_ | -32 (-55 to -8) | 0.009 | -32 (-56 to -8) | 0.01 |
| Vehicle km driven per year within 100m from school | FVC | -2 (-13 to 8) | 0.65 | -3 (-13 to 8) | 0.62 |
|  | FEV_1_ | 0 (-8 to 9) | 0.96 | 0 (-9 to 9) | 0.99 |
|  | FEF_25_ | 4 (-21 to 29) | 0.75 | 6 (-19 to 32) | 0.63 |
|  | FEF_50_ | 1 (-17 to 18) | 0.95 | 1 (-17 to 18) | 0.95 |
|  | FEF_75_ | 2 (-8 to 12) | 0.69 | 2 (-8 to 11) | 0.77 |
