# Supplementary material for: Long-Term Exposure to Primary Traffic Pollutants and Lung Function in Children: Cross-Sectional Study and Meta-Analysis
Source: PLoS One. 2015 Nov 30;10(11):e0142565. doi: 10.1371/journal.pone.0142565 (PMC4664276; doi:10.1371/journal.pone.0142565)
Supplement: S5 Table — (DOCX) [file pone.0142565.s011.docx]

S5 Table. Number of participants with missing data for each variable of interest

| **Independent variable** | **Missing values** |
| --- | --- |
| NO_2_ (µg/m^3^) | 0 (0%) |
| NO (µg/m^3^) | 0 (0%) |
| NO_x_ (µg/m^3^) | 0 (0%) |
| O_3_ (µg/m^3^) | 0 (0%) |
| Oxidants (µg/m^3^) | 0 (0%) |
| PM_10_ (µg/m^3^) | 0 (0%) |
| PM_10_ exhaust (µg/m^3^) | 0 (0%) |
| PM_10_ non exhaust (µg/m^3^) | 0 (0%) |
| PM_2.5_ (µg/m^3^) | 0 (0%) |
| PM_2.5_ exhaust (µg/m^3^) | 0 (0%) |
| PM_2.5_ non exhaust (µg/m^3^) | 0 (0%) |
| Pm coarse (µg/m^3^) | 0 (0%) |
| Distance of home from highly trafficked roads (meters) | 0 (0%) |
| Vehicle km driven per year within 100m from home | 0 (0%) |
| Distance of school from highly trafficked roads (meters) | 0 (0%) |
| Vehicle km driven per year within 100m from school | 0 (0%) |
| Month of measurement | 0 (0%) |
| trunk length | 0 (0%) |
| ethnic subgroup | 0 (0%) |
| observer | 0 (0%) |
| sex | 0 (0%) |
| age | 1 (<0.1%) |
| indoor room temperature | 43 (0.8%) |
| school | 0 (0%) |
| cotinine | 127 (2.6%) |
| IMD score | 1 (<0.1%) |
| NS-SEC group | 39 (0.8%) |
| sum of skin folds | 14 (0.3%) |
| fat mass index | 78 (1.6%) |
