# Supplementary material for: Long-Term Exposure to Primary Traffic Pollutants and Lung Function in Children: Cross-Sectional Study and Meta-Analysis
Source: PLoS One. 2015 Nov 30;10(11):e0142565. doi: 10.1371/journal.pone.0142565 (PMC4664276; doi:10.1371/journal.pone.0142565)
Supplement: S6 Table — (DOCX) [file pone.0142565.s012.docx]

S6 table. Additional information on the studies included in the meta-analysis.

| **Author** | **Level of the exposure assessment** | **Confounders included in the final model** |
| --- | --- | --- |
| Peters 1999 | Community level | Age, sex, height, weight, ethnicity, reported asthma, parental smoking, pets at home, gas stove |
| Oftedal 2008 | Lifetime residential exposure | Age, sex, height, BMI, birth weight, temperature lags 1-3 days before spirometry, current asthma, parental smoking, ethnicity, parental education |
| Dales 2008 | Current residential exposure | Age, sex, height, weight, ethnicity, passive smoking at home, pets at home, acute respiratory illness or medication for asthma in the preceding 2 weeks |
| Rosenlund 2009 | Current residential exposure | Age, sex, height, weight. |
| Lee 2011 | Community level | Age, sex, height, weight. |
| Svendsen 2012 | Current residential exposure | Age, sex, height, weight, ethnicity, parental education, passive smoking at home, school elevation |
| Gehring 2013 (BAMSE, Gini-Lisa, Gini South, MAAS, PIAMA | Current residential exposure | Age, sex, height, weight, recent respiratory infections, ethnicity, parental education, parental history of allergy, breast feeding, mother smoking during pregnancy, passive smoking, mold/dampness at home, pets at home |
| Urman 2013 | Current residential exposure | Age, sex, height, weight, BMI, ethnicity, respiratory illness, field technician, community |
| Morales 2015 | Current residential exposure | Age, sex, height, weight, area of study, ethnicity |
| CHASE | Current residential exposure | Age, sex, trunk length, month of measurement, ethnic subgroup, observer, indoor room temperature |
